# Symbiosis islands of Loteae-nodulating *Mesorhizobium* comprise three radiating lineages with concordant *nod* gene complements and nodulation host-range groupings

**DOI:** 10.1099/mgen.0.000426

**Published:** 2020-08-26

**Authors:** Benjamin J. Perry, John T. Sullivan, Elena Colombi, Riley J.T. Murphy, Joshua P. Ramsay, Clive W. Ronson

**Affiliations:** ^1^​ Department of Microbiology and Immunology, University of Otago, Dunedin, New Zealand; ^2^​ School of Pharmacy and Biomedical Science, Curtin University, Perth, Australia

**Keywords:** *Mesorhizobium*, *Lotus*, Loteae, nodulation, host range, symbiosis, ICE, evolution

## Abstract

*
Mesorhizobium* is a genus of soil bacteria, some isolates of which form an endosymbiotic relationship with diverse legumes of the Loteae tribe. The symbiotic genes of these mesorhizobia are generally carried on integrative and conjugative elements termed symbiosis islands (ICESyms). *Mesorhizobium* strains that nodulate *Lotus* spp. have been divided into host-range groupings. Group I (GI) strains nodulate *L. corniculatus* and *L. japonicus* ecotype Gifu, while group II (GII) strains have a broader host range, which includes *L. pedunculatus*. To identify the basis of this extended host range, and better understand *Mesorhizobium* and ICESym genomics, the genomes of eight *Mesorhizobium* strains were completed using hybrid long- and short-read assembly. Bioinformatic comparison with previously sequenced mesorhizobia genomes indicated host range was not predicted by *Mesorhizobium* genospecies but rather by the evolutionary relationship between ICESym symbiotic regions. Three radiating lineages of Loteae ICESyms were identified on this basis, which correlate with *Lotus* spp. host-range grouping and have lineage-specific *nod* gene complements. Pangenomic analysis of the completed GI and GII ICESyms identified 155 core genes (on average 30.1 % of a given ICESym). Individual GI or GII ICESyms carried diverse accessory genes with an average of 34.6 % of genes unique to a given ICESym. Identification and comparative analysis of NodD symbiotic regulatory motifs – *nod* boxes – identified 21 branches across the NodD regulons. Four of these branches were associated with seven genes unique to the five GII ICESyms. The *nod* boxes preceding the host-range gene *nodZ* in GI and GII ICESyms were disparate, suggesting regulation of *nodZ* may differ between GI and GII ICESyms. The broad host-range determinant(s) of GII ICESyms that confer nodulation of *L. pedunculatus* are likely present amongst the 53 GII-unique genes identified.

## Data Summary

Sequencing data and genome assemblies are deposited in NCBI SRA and Genome databases, bioproject: PRJNA496338. The hybrid assembly pipeline used is available at https://github.com/BenjaminJPerry/HybridAssembly. Three supplementary figures and four supplementary tables are included in the online version of this article.

**Impact Statement**

The symbiotic partnership between rhizobia and legumes provides a primary conduit for elemental nitrogen to enter the global food web. This symbiotic interaction has evolved strict signalling requirements from both the plant and bacterial perspectives. In the *Mesorhizobium-Lotus* symbiosis the bacterial symbiotic genes are horizontally acquired in nature via an integrative and conjugative element termed a symbiosis island. Some symbiosis islands confer differing symbiotic compatibility with *Lotus* spp. Here we identified an evolutionary relationship between symbiosis islands, independent of the *Mesorhizobium* chromosomes that harbour them, which correlated with the observed host-range differences and unique *nod* gene complements they confer. We further identified differences in their symbiotic gene regulation and accessory gene complements which may explain the expanded host range conferred by one of these symbiosis island lineages.

## Introduction

The genus *Mesorhizobium* encompasses a group of ubiquitous saprophytic soil bacteria. Mesorhizobia can evolve the ability to engage in nitrogen-fixing endosymbiosis with leguminous plants of the genus *Lotus* via the horizontal acquisition of a genetic element termed a symbiosis island [1, 2]. Symbiosis islands (ICESyms) are a specific phenotypic category of integrative and conjugative element, which carry the genes necessary for symbiotic signalling and nitrogen fixation [3–5]. ICESyms have been shown to transfer in the environment, resulting in the acquisition of symbiotic potential by native *Mesorhizobium* populations [1, 2, 6]. ICESym fitness is ultimately dictated by the success of the mesorhizobia in which it resides in the soil microbial community. ICESym transfer to saprophytic mesorhizobia followed by clonal expansion within root nodules and subsequent release following nodule senescence likely results in an overall increase in ICESym abundance in the soil [7]. In the context of agriculture ICESym transfer may be detrimental as native mezorhizobia that receive an ICESym are not always effective symbionts but can outcompete effective inoculant strains for nodulation [6].

The symbiotic compatibility of *Mesorhizobium* strains and *Lotus* species is mediated by molecular signalling between the two partners [8, 9]. This signalling is initiated by the perception of plant root-derived flavonoids by the bacterial regulatory protein NodD [10]. NodD is a LysR family transcriptional regulator, which binds a defined 47 bp DNA motif, referred to as a *nod* box, located upstream of *nod* genes [11]. When the activating flavonoid molecule is absent, NodD bound to *nod* boxes in some cases represses expression of downstream genes [12–14]; upon binding of a compatible plant flavonoid, NodD undergoes a conformational change, which results in enhanced binding affinity to the *nod* box as well as induction of downstream *nod* genes [15, 16]. The *nod* genes induced by activated NodD encode enzymes required for the synthesis of lipo-chitooligosaccharide signalling molecules termed Nod factor (NF) [17]. NF is composed of a chitin oligomer backbone with a fatty acid at the non-reducing end. Molecular decorations added to the chitin oligomer at specific locations, and variation in the length and degree of saturation of the fatty acid ‘tail’, confer strain-specific diversity to NF, which can dictate symbiotic host range [18]. In addition to primary NF signalling, bacterial effector proteins transported by type I, III, IV, VI secretion systems [19–26], and additionally polysaccharides [27–33], have been implicated in further modulating symbiotic signalling.

The genus *Lotus* is within the Loteae tribe of legumes, which includes several other genera such as *Acmispon*, *Anthyllis*, *Ornithopus* and *Scorpiurus* [34]. *Lotus* spp. are distributed globally and occupy seven taxonomic clades, within which the *L. corniculatus* group (including *L. japonicus*) and *L. pedunculatus* group occupy two distinct subgroups within clade B [35, 36]. Interest in *Lotus* spp. in New Zealand originated from the potential use of *L. corniculatus* or *L. pedunculatus* as perennial pasture legumes in infertile hill-country soils [37]. This resulted in the accumulation of diverse *Mesorhizobium* strains in the NZP culture collection by researchers at The Department of Scientific and Industrial Research, Palmerston North, New Zealand [38–41]. Subsequently, *L. japonicus* ecotype Gifu, a diploid relative of *L. corniculatus*, was adopted as a model legume for the study of molecular genetics and physiology because of specific life history and genetic traits that make it amenable to laboratory study [42]. Early bacteriological work in New Zealand with *Lotus*-nodulating *Mesorhizobium* strains of *L. corniculatus* and *L. pedunculatus* identified two phenotypic groups of strains: group I (GI) strains, which form effective nodules on the *L. corniculatus* subgroup while only inducing nodule primordia on *L. pedunculatus*; and group II (GII) strains, which effectively nodulate both the *L. corniculatus* subgroup and *L. pedunculatus* [40]. Subsequently, studies of the GII strain NZP2037 identified its ability to nodulate several *Lotus* species, *Ornithopus sativus*, *Leucaena leucocephala* and the New Zealand native legumes *Carmichaelia flagelliformis* and *Clianthus puniceus*; all of which were not nodulated by GI strains [43]. Hence NZP2037 is now considered to be a broad-host-range strain [44].

In this work we completed the genome sequences of multiple *Lotus*-nodulating mesorhizobia and investigated the taxonomic, structural and genetic similarities of their ICESyms to better understand the evolutionary origins of their ICESyms and host ranges. Additionally, through pangenomic analysis of the completed GI and GII ICESym sequences, we identified candidates for the genetic basis of the broad host range conferred by GII ICESyms.

## Methods

### Bacterial strains and culture conditions

Bacterial strains are described in Table 1. *Mesorhizobium* strains were grown on G/RDM solid medium [45] containing 25 ug ml^−1^ fosfomycin or, for DNA extractions, in tryptone-yeast extract (TY) broth containing 25 ug ml^−1^ fosfomycin with shaking at 28 °C.

**Table 1.** Comparison of GI and GII *Mesorhizobium* genomes and ICESyms

Species	Strain designation	Synonymous strain ID	Geographic origin	*Lotus* spp. nodulation host range	Host-range group	ONT coverage	Illumina coverage	Genome size (bp)	Genome GC%	ICESym structure	ICESym size (bp)	ICESym GC%	Strain original publication	Prior genome-sequencing data	GenBank accession
* M. japonicum *	R7A	Reisolate of: ICMP 3153; NZP2238; Lc265Da	New Zealand (Ireland)	*L. corniculatus,* *L. japonicus* Gifu	GI	27	490	6 529 994	62.9	Monopartite	501 815	59.3	Sullivan *et al.* (1995) [1]	Kelly *et al.* (2014) [69]	CP033366.1
*M.* sp*.*	R88B	ICESym-Transconjugate: ICMP 3153	New Zealand	*L. corniculatus,* *L. japonicus* Gifu	GI	36	361	7 207 166	62.4	Monopartite	501 815	59.3	Sullivan *et al.* (1995) [1]	Reeve *et al.* (2014) [70]	CP033367.1
* M. japonicum *	MAFF303099	na	Japan	*L. corniculatus,* *L. japonicus* Gifu	GI	na	na	7 596 297	62.7	Monopartite	611 008	59.7	Kaneko *et al.* (2000) [68]	na	BA000012.4, BA000013.4, AP003017.1
*M.* sp*.*	NZP2234	ICMP 10866; CC811	United States	*L. corniculatus,* *L. japonicus* Gifu	GI	19	37	6 749 717	63.1	Monopartite	422 420	59.6	Bailey *et al.* (1971) [38]	na	CP033364.1
*M.* sp*.*	NZP2298	ICMP 12619	Canada	*L. corniculatus,* *L. japonicus* Gifu	GI	50	40	7 336 816	62.8	Monopartite	467 156	59.7	Charlton (1981) [39]	na	CP033365.1
* M. erdmanii *	NZP2014	ICMP 10717	New Zealand	*L. corniculatus,* *L. japonicus* Gifu*,* *L. pedunculatus*	GII	38	126	6 602 217	62.9	Monopartite	528 908	59.2	Bailey *et al.* (1971) [38]	Haskett *et al.* (2016) [54]	CP033361.1
*M.* sp*.*	NZP2042	ICMP 10765	New Zealand	*L. corniculatus,* *L. japonicus* Gifu*,* *L. pedunculatus*	GII	24	136	6 870 350	63.1	Tripartite	526 858	59.6	Bailey *et al.* (1971) [38]	Haskett *et al.* (2016) [54]	CP033334.1
* M. loti *	NZP2037	ICMP 1326	New Zealand	*L. corniculatus,* *L. japonicus* Gifu*,* *L. pedunculatus,* *L. divaricatus*	GII	na	na	7 481 739	62.9	Tripartite	562 308	59.4	Bailey *et al.* (1971) [38]	Haskett *et al.* (2016) [54]	CP016079.1, CP016080.1
* M. loti *	SU343	ICMP 10808; NZP2196	United States	*L. corniculatus,* *L. japonicus* Gifu*,* *L. pedunculatus*	GII	21	120	7 200 913	63.0	Tripartite	556 132	59.4	Crow *et al.* (1981) [40]	Haskett *et al.* (2016) [54]	CP033368.1, CP033369.1, CP033370.1
* M. jarvisii *	ATCC 700743^T^	ICMP 4682; ATCC 33669^T^	na	*L. corniculatus,* *L. japonicus* Gifu*,* *L. pedunculatus*	GII	16	139	7 201 057	63.0	Tripartite	556 132	59.4	Martinez-Hidalgo *et al.* (2015) [71]	na	CP033507.1, CP033508.1, CP033509.1

### DNA isolation for sequencing

For Illumina sequencing, gDNA was extracted from stationary-phase TY cultures using a Qiagen DNeasy UltraClean Microbial Kit. For sequencing on an Oxford Nanopore Technologies (ONT) MinION, high-molecular-weight gDNA was extracted as previously described [46].

### Library preparation and sequencing

Long-read (LR) sequencing libraries were prepared using a Rapid Barcoding Kit (ONT, SQK-RBK004) using 500 ng of high-molecular-weight DNA. Sequencing was carried out on the MinION sequencer using a FLO-MIN106 flow cell containing v9.4 nanopores. Libraries were sequenced for a total of 48 h, with two restarts using fresh libraries at hours 19 and 22.5 of sequencing.

Illumina sequencing libraries were generated using a Nextera XT Library Prep Kit (Illumina) and sequenced with an Illumina MiSeq benchtop sequencer using 600-bp v3 chemistry. Illumina data available for previously sequenced genomes were downloaded from the NCBI Sequence Read Archive (SRA). All sequencing data were deposited into the NCBI SRA under the bioproject PRJNA496338.

### Genome assembly and annotation

The LRs were base-called in fastq format using Albacore v2.1.1, and adapters were trimmed using Porechop v0.2.3 (https://github.com/rrwick/Porechop) with default settings. Quality trimming of LRs was conducted using Filtlong v0.2.0 (https://github.com/rrwick/Filtlong) discarding the lowest quality 20 % of the data and reads less than 1000 bp in length. Corresponding paired-end reads (PRs) were quality trimmed to minimum Q20, then *k*-mer error corrected using SPAdes v3.12 [47, 48]. The *k*-mer corrected PRs were then used to *k*-mer error correct the filtered LRs using LoRDEC v 0.9 [49]. The *k*-mer corrected LRs were used for *de novo* genome assembly using Flye v2.3.5 [50], and the subsequent genome assembly graphs were passed to Unicycler v0.4.6 [51], in conjunction with the trimmed PRs and *k*-mer corrected LRs, for final hybrid assembly and consensus improvement. This hybrid assembly pipeline utilizes *de novo* assemblies of both Oxford Nanopore LRs and Illumina PRs, followed by merger and consensus correction. The use of *k*-mer-based error correction of LRs prior to assembly allows for correction of regions which may suffer from multiple alignment penalties in alignment-based consensus correction methods. *De novo* assembly using *k*-mer corrected LRs also maximizes the utility of LR data. The hybrid assembly pipeline with documentation for installation and usage can be found in the GitHub repository: https://github.com/BenjaminJPerry/HybridAssembly.

The start position of all assembled genomes and plasmid replicons was adjusted to *dnaA* or *repA* homologues respectively with Circulator fixstart v1.5.5 [52] using the *dnaA* or *repA* accessions from the NZP2037 or MAFF303099 genomes (A9174_RS00005, A9174_33635, mll5581, mll9353, and mll9654). The completed assemblies were annotated using the Prokaryotic Genome Annotation Pipeline [53]. For pangenomic comparison of ICESyms, ICESym sequences were excised from the genome using the *att* site boundaries and the locations of the ICESym-associated integrases based on previously described ICESym annotations [2, 54]. Annotation of the excised ICESym sequences was performed using Prokka v1.13.3 [55] using a *Mesorhizobium *-specific annotation database constructed from the NCBI annotations of all genomes compared in this work.

### Analysis of locally colinear blocks

Whole-genome and ICESym structural comparisons were conducted using locally colinear blocks (LCBs) calculated and visualized using Mauve [56]. Analysis of locally colinear DNA sequence conserved across ICESyms used the alignment intervals reported in the Mauve backbone output file by filtering for regions conserved across all ICESyms.

### Hidden Markov model analysis

The *nod* box and NifA motifs were identified using nhmmscan from hmmer 3 [57, 58]. A training set of previously characterized *nod* box or NifA-binding sequences from the R7A and NZP2037 ICESyms [3, 4] were used to generate HMMs. The *nod* box motifs were extracted and a maximum-likelihood tree with 1000 bootstraps was constructed using mega 7 [59]. *Nod* box clades identified by phylogenetic analysis were then assigned nomenclature based on the first coding sequence proximal to the end of the *nod* box motif.

### Pangenome analysis

**Fig. 1.**
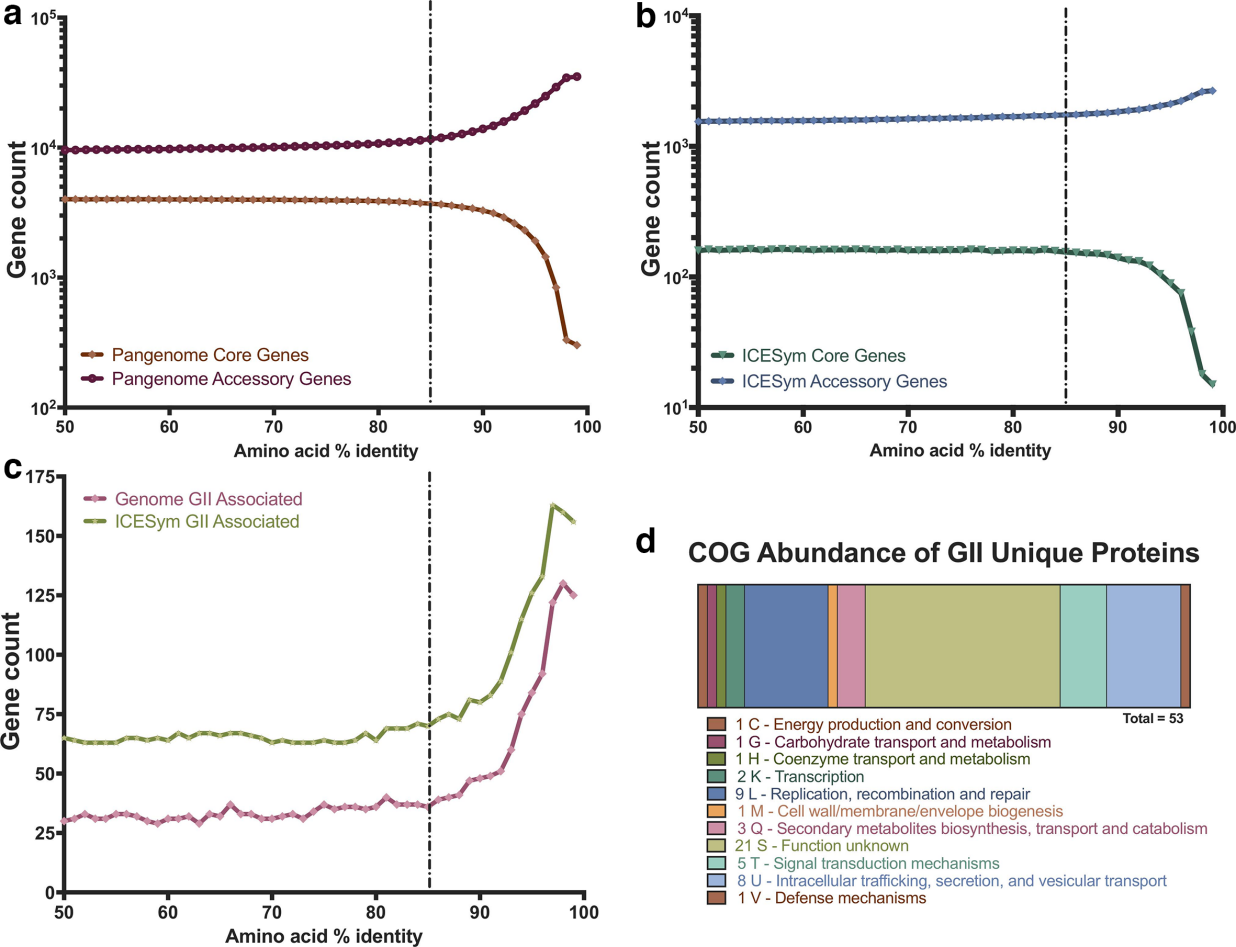
Summary of GI and GII pangenomic comparison and GII-ICESym uniquely conserved genes. (a) Pangenome calculation of GI and GII core and accessory genes as a function of the AAi thresholds used. (b) Pangenome calculation of core and accessory genes within GI and GII ICESym regions as a function of AAi threshold used. (c) Total uniquely conserved GII-ICESym genes across genomes versus ICESyms as a function of AAi threshold used for pangenome calculations. (d) Abundance of COG single-letter functional groups annotated to GII-ICESym uniquely conserved genes.

Pangenome and pan-ICESym calculations used the Roary pangenome analysis pipeline and the ten complete *Mesorhizobium* genomes (Table 1) [60]. Initially, pangenome calculations were replicated using iterative incrementation of the cut-off for blastP assignment of protein ortho-groups (-i) from 50–99 % amino-acid identity (AAi) (Fig. 1). Identification of GII-genome-unique ortho-groups was then conducted using Scoary [61], incrementing over each iteration of the Roary pangenome calculation. Pangenome analysis of ICESyms was conducted using the Prokka-annotated ICESyms (previously described); pangenome and GII-ICESym unique ortho-groups were calculated iteratively as previously described. Additional functional annotation information was appended to ICESym-Core and GII-ICESym unique protein coding sequence using EggNOG mapper [62, 63]. Proteins without predicted COG functional categories were assigned to ‘category S – unknown function’.

### Analysis of average nucleotide identity

To identify *Mesorhizobium* strains with the potential to nodulate *Lotus* spp. for ANI comparison, we downloaded the 1077 *Mesorhizobium* genomes from the NCBI Database and the 163 *Mesorhizobium* genomes maintained in the JGI IMG database and used these to build a blastn [64] database (accessed on 23 April 2020). We queried the genomes using blastn for a copy of the key host-range gene *nodZ* [8] and collected the genomes with a *nodZ* blastn result E-value of ≤1.56E-11 to the *nodZ* sequence of SU343, after which there was an obvious drop in match quality. This identified 90 unique *Mesorhizobium* genomes. To collect the regions within these assemblies which represented the ICESym symbiotic regions, we used a combination of in-house bash scripts to split the contigs of a genome assembly at all known ICESym *attB* integration sites, and then collected the contigs, which contained *nod* box or NifA motifs identified by HMM analysis.

We then screened the 90 *nodZ* containing genomes to find those likely to perceive *Lotus* symbiotic signalling molecules, by calculating a pangenome for the ICESym symbiotic regions of the 90 genomes and identifying those which contained homologues of both NodD1 and NodD2 with the ten GI and GII *Mesorhizobium* (Table 1) genomes at 85 % AAi or greater. This resulted in identification of 43 *Mesorhizobium* genomes, which contained NodZ, NodD1 and NodD2. Average nucleotide identity was calculated using fastANI [65] between the 43 genomes and their ICESym symbiotic regions. A window size of 1000 bp and minimum pairwise fragment coverage of 20 % were used for both genomic and ICESym symbiotic regions comparisons. For visualization, ANI distance matrices were plotted using R v3.6.3 [66] and the heatmaply package [67].

## Results and discussion

### Completion of eight *Lotus-*nodulating *Mesorhizobium* genomes using hybrid assembly

Ten *Mesorhizobium* strains with well-defined nodulation host ranges were selected for detailed functional genomic comparison: five GI strains that nodulate *L. corniculatus* but only formed uninfected nodule primordia on *L. pedunculatus*, and five GII strains that nodulate both *L. corniculatus* and *L. pedunculatus* effectively (Table 1). To conduct the genomic comparison exhaustively we compared only completed genomes sequences to facilitate delineation of complete ICESym regions. Prior to the initiation of this work, fully completed genomes existed for only two *Mesorhizobium* strains with well-characterized host ranges that fit within the GI or GII host-range framework: MAFF303099 [68] and NZP2037 [54]. Scaffold-quality genome assemblies were also available for five strains: R7A [69], R88B [70], NZP2014, NZP2042 and SU343 [54]; while the genomes of *M.* sp. NZP2234, *M.* sp. NZP2298 and *M. jarvisii* ATCC 700743^T^ (formerly ATCC 33669^T^) [71] had not been sequenced. A hybrid assembly approach combining Oxford Nanopore long-read sequencing data with Illumina paired-end sequencing data was used. This resulted in the completion of the five draft and three novel genomes, yielding ten complete *Lotus-*nodulating *Mesorhizobium* genomes from diverse geographic origins for detailed comparison (Table 1).

Sizes of the eight newly completed genomes ranged from 6.5 to 7.3 Mb, while their ICESyms ranged from 422 to 562 kb. The GC content of the genomes varied from 62.4–63.1% and the ICESyms from 59.2–59.7 % (Table 1). The chromosomes of the completed genomes appeared generally syntenic, with the exception of tripartite ICESym-containing strains (NZP2042, SU343, NZP2037 and ATCC 700743^T^). These strains have a 480 kb chromosomal inversion and translocation, flanked by the *beta* and *gamma* fragments of the tripartite ICESym (Fig. S1, available in the online version of this article), resulting from the tripartite integration mechanism [54]. NZP2298 contained four tRNA genes not found in the other strains that were located on the chromosome within a putative 35 kb prophage. Strains SU343 and ATCC 700743^T^ each contained one large plasmid and a 24 kb putative extra-chromosomal plasmidial prophage within their genomes. This circular 24 kb DNA molecule encoded phage-like proteins, a toxin-antitoxin system, a putative two-gene restriction/modification system, a XreC/D recombinase, and a Y-family DNA polymerase. Analysis of depth of coverage of Illumina reads indicated the putative plasmidial prophage replicon was present at 0.93–2.93× coverage relative to the chromosomes in the ATCC 700743^T^ and SU343 assemblies, suggesting it was maintained at two copies per cell.

### The genomes of NZP2037, SU343 and ATCC 700743^T^ are near-isogenic

The entire genomes of isolates *M. loti* SU343, and *M. jarvisii* ATCC 700743^T^ were isogenic, with only 47 SNPs, identified by Mauve alignment, distinguishing them. *M. jarvisii* ATCC 700743^T^ was described as a result of work that confirmed the ATCC and USDA culture collections contained *M. loti* NZP2213^T^ type strain accessions, which did not correspond to the original biomaterial. One of these accessions, isolate ATCC 33669^T^, was revealed as a mixed culture of what are now strains *M. jarvisii* ATCC 700743^T^ and *M. erdmanii* USDA 3471^T^ [71] and neither strain was in fact *M. loti* NZP2213^T^. The strains SU343 and ATCC 700743^T^ were also highly similar to NZP2037, but the chromosome of NZP2037 contained multiple unique insertions and deletions (Fig. S1). Two of the unique insertions were greater than 100 kb and found adjacent to tRNA genes. Additionally, *M. loti* NZP2037 contained a 474 kb plasmid pRlo2037, while SU343 and ATCC 700743^T^ both contained a 242 kb plasmid with a highly similar backbone to pRlo2037. pRlo2037 contained six regions absent from the plasmids of SU343 and ATCC 700743^T^.

### *Mesorhizobium* taxonomy does not predict host range due to horizontal transfer of ICESyms between genospecies

A taxonomic comparison of GI and GII *Mesorhizobium* genomes with other confirmed and putative *Lotus*-nodulating *Mesorhizobium* was completed using *in silico* DNA–DNA hybridization. Putative *Lotus-*nodulating mesorhizobia were identified based on the presence of NodZ and homologues of both NodD1 and NodD2 shared by the GI and GII *Mesorhizobium* (Table 1). NodZ was chosen as a primary marker for *Lotus* symbiotic compatibility as previous studies have implicated it in symbiotic signalling in *Mesorhizobium* [8]. It is also present in a *Bradyrhizobium* sp. WM9, which nodulates *Lotus* [72, 73], and heterologous expression of *nodZ* and *nolL* from *B. japonicum* in *R. leguminosarum* conferred nodulation of some *Lotus* spp. [74].

**Fig. 2.**
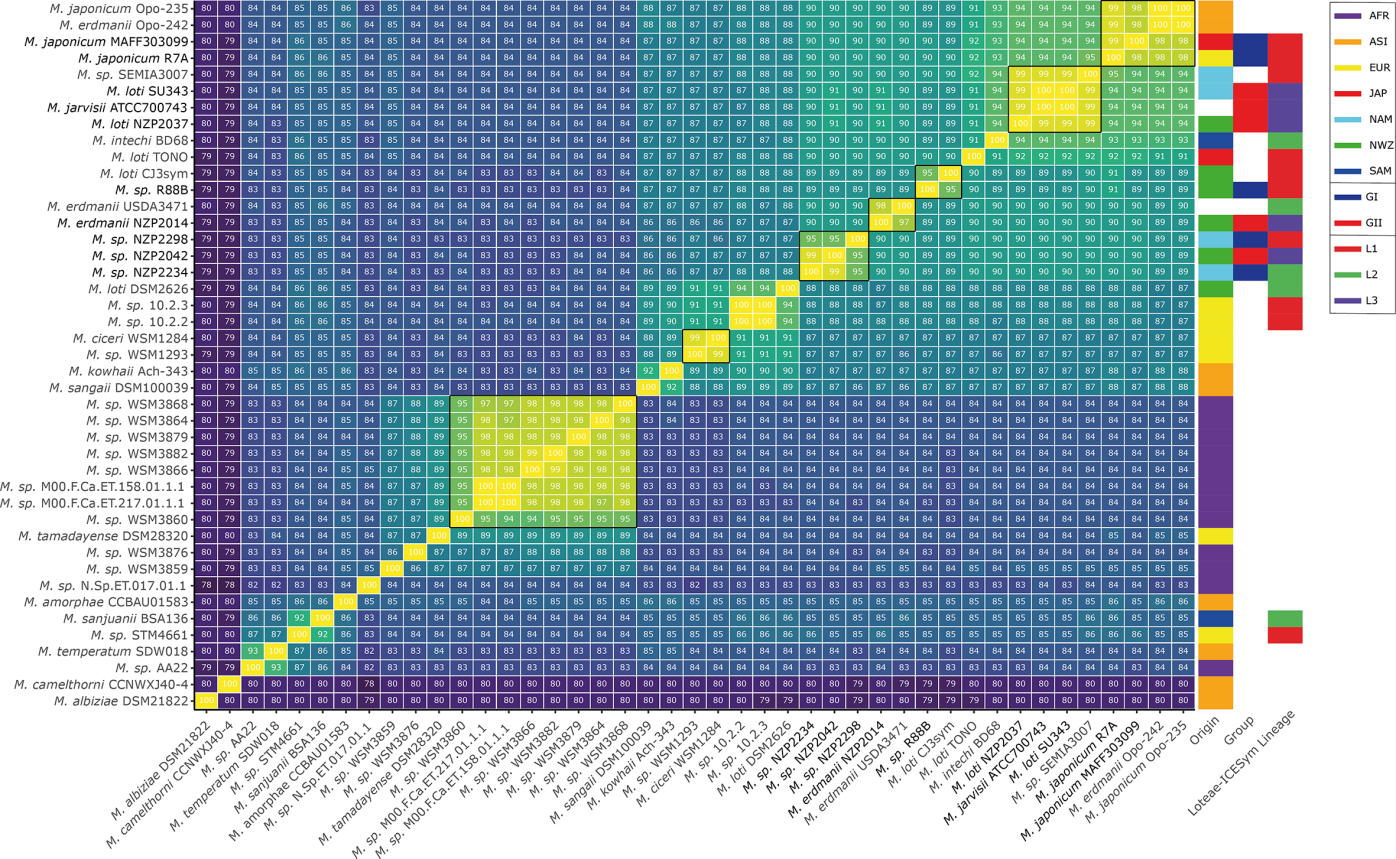
Average nucleotide identity of *Mesorhizobium* genomes containing NodZ, NodD1 and NodD2 homologues. *Mesorhizobium* genomes were selected based on the conservation of homologues of all three of NodZ, NodD1 and NodD2 of the GI and GII strains at 85 % AAi or greater. Putative genospecies with ANI >95 % are grouped with black borders. Origin indicates the region a strain was originally isolated from: AFR – Africa, ASI – Asia, EUR – Europe, JAP – Japan, NAM – North America, SAM – South America. L1 – Lineage 1 Loteae ICESyms, L2 – Lineage 2 Loteae ICESyms, L3 – Lineage 3 Loteae ICESyms.

ANI scores of 95–96 % are considered to demarcate the species (genospecies) boundary in rhizobia and other micro-organisms [75]. The ten GI and GII strains comprised four putative genospecies at ANIs of ≥95 % (Fig. 2), with *M. jarvisii* ATCC 700743^T^, *M. loti* NZP2037 and *M. loti* SU343 belonging to the same genospecies. *M.* sp. SEMIA 3007 also belonged to this genospecies. The strains *M.* sp. R88B and *M.* sp. CJ3sym are derived from New Zealand soils [1, 2], and represent a unique genospecies. *M.* sp NZP2298, *M.* sp. NZP2234 and *M.* sp. NZP2042 represent yet another novel *Mesorhizobium* genospecies that is distributed globally. Our ANI analysis revealed that while GI and GII strains occupy a sub-clade within a range of 89–100% ANI, other known symbionts of *Lotus* spp. such as *M. loti* DSM 2626^T^ (a synonymous accession of NZP2213^T^=ICMP 4682^T^ [41]), *M. ciceri* WSM1284 [76] and *M. sanjuani* BSA136^T^ [77], are scattered through a continuum of *Mesorhizobium* genospecies (Fig. 2).

The GI and GII strains segregated into separate genospecies with the exceptions of NZP2234 (GI), NZP2298 (GI) and NZP2042 (GII), which shared a genomic ANI of 95–99 % (Fig. 2; File S1 ANI Genomes.html). Additionally, the GI and GII genospecies did not segregate into distinct evolutionary lineages but were intermingled amongst each other. This indicates that nodulation host range cannot be predicted merely by taxonomic identification of a *Mesorhizobium* strain. Furthermore, the observed disconnect between taxonomic relatedness of mesorhizobia and host range implies that horizontal transfer of ICESyms occurs between distinct genospecies, consistent with previous studies examining ICESym transfer in the environment [1, 6].

### GI and GII ICESyms have shared synteny indicative of common ancestry

**Fig. 3.**
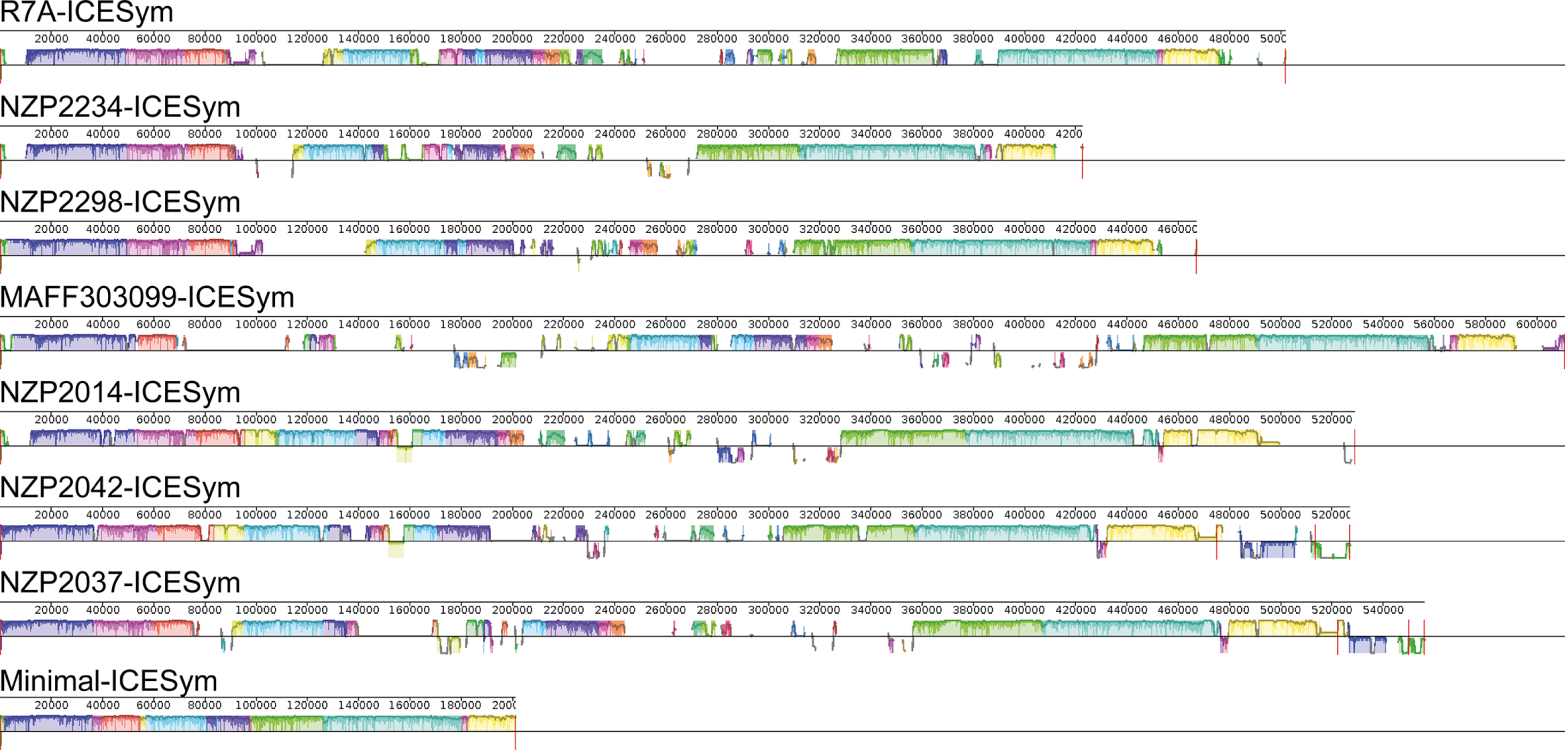
Shared synteny of non-isogenic GI and GII ICESyms. Coloured regions indicate LCBs across ICESyms. Colourless stretches indicate ICESym unique regions, and regions below the axis indicate regions inverted relative to the R7A ICESym. For tripartite ICESyms, boundaries of the α, β and γ fragments are indicated with vertical red lines. The Minimal-ICESym maintains the conserved order of LCBs shared by all ICESyms.

Syntenic comparison of the seven non-isogenic GI and GII ICESyms using Mauve identified 111 conserved regions used to compute 14 LCBs common across these ICESyms, which cumulatively represented a theoretical Minimal-ICESym (Fig. 3). The 14 LCBs represented 201 036 bp, which equates to 39.4 % (±4.8 % sd) of an ICESym’s sequence on average. When the order of the 14 LCBs was compared, shared synteny was observed with the 14 LCBs arranged into two conserved blocks interspersed with ICESym-unique regions as previously suggested [3, 4]. The conserved order of the 14 LCBs in both GI and GII ICESyms suggests that they arose from a common ancestor and have diverged via horizontal acquisition and exchange of genetic information in distinct physical units. Recently, a comparison of *Mesorhizobium* ICESyms captured in *Cicer* spp. nodules suggested a similar arrangement of conserved genes [78].

### GI and GII ICESyms are distributed across three radiating lineages of Loteae ICESyms

**Fig. 4.**
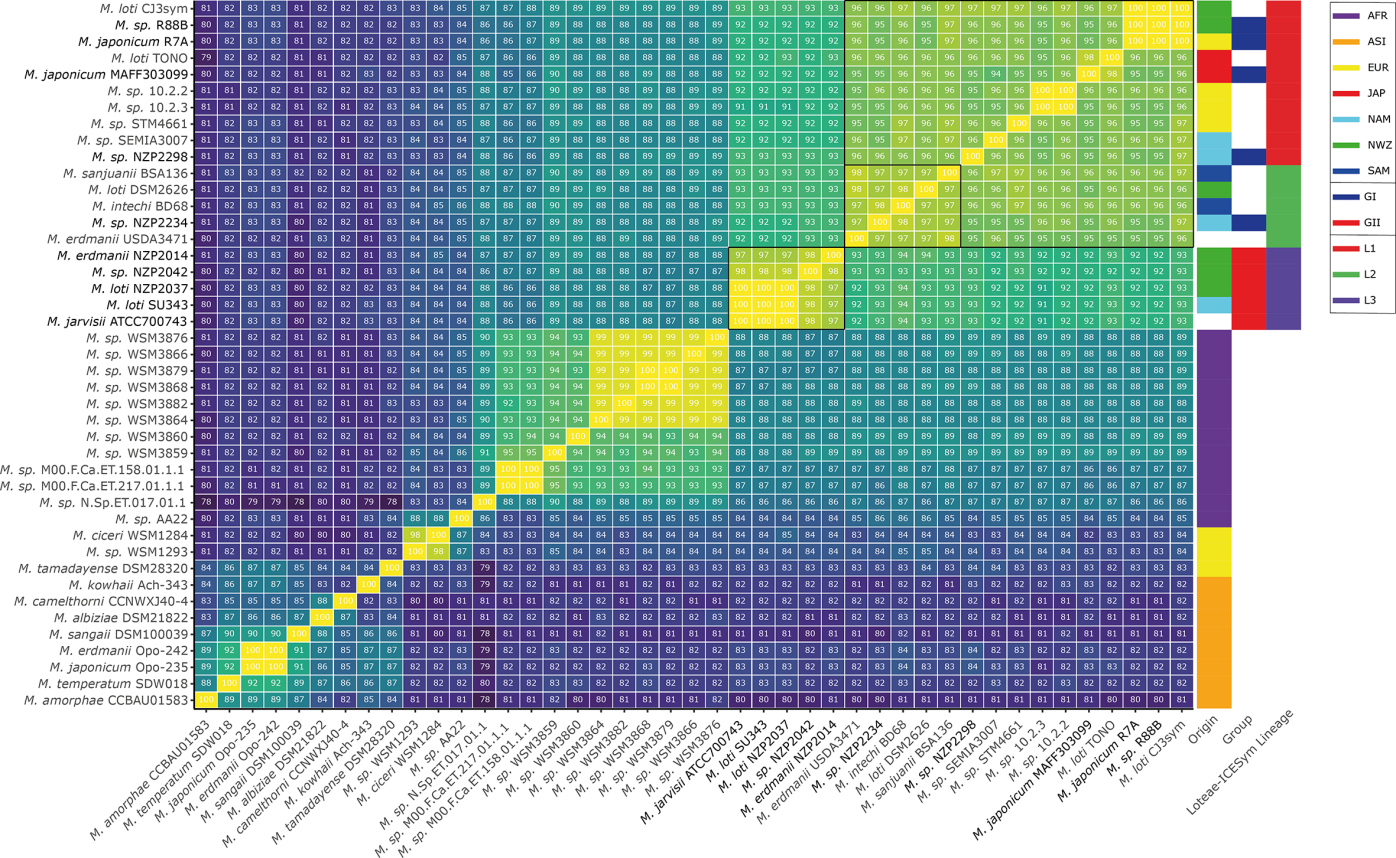
Average nucleotide identity of ICESym symbiotic regions. ANI comparison of ICESym symbiotic regions extracted from the 43 *Mesorhizobium* strains shown in Fig. 2. Symbiotic regions were identified based on the presence of a *nod* box or *nifA* binding motif within an assembled contig, and genomic DNA was trimmed from contigs using ICESym integrase *attP* sites. Pairwise comparisons of the 43 ICESym symbiotic regions spanned an average of 175 949 bp (±74 537 bp sd). Groupings representing Loteae ICESym lineages are indicated with black borders.

Evolutionary comparison of 43 ICESym symbiotic regions from *Mesorhizobium* genomes which contained homologues of GI/GII NodZ, NodD1 and NodD2 was calculated using fastANI [65]. As mobile genetic elements such as ICESyms do not have species, applying the 95 % ANI species demarcation threshold may be incorrect and as such we interpret the ANI of the ICESym symbiotic regions based on their self-evident groupings and *nod* gene complements. The extracted sequences of ICESym symbiotic regions had an average cumulative size of 453 797 bp (±195 788 bp sd), which was comparable to the sizes of the complete ICESyms in GI and GII strains (Table 1). ANI comparison of the 43 ICESym symbiotic regions identified three lineages of ICESyms within *Mesorhizobium* isolated from legumes of the Loteae tribe, which shared ANI of 91 % or greater (Fig. 4). Lineage 1 (L1) had the largest representation with ICESyms which shared ANI values of 94 % or greater, and included the ICESyms of GI strains NZP2298, MAFF303099, R88B and R7A (Fig. 4). Lineage 2 (L2) appeared as a group of closely related Loteae ICESyms with ≥97 % ANI, within a larger group which encompasses the L1 Loteae ICESyms. L2 included the ICESym symbiotic regions of the GI strain *M.* sp. NZP2234, as well as those of *M. erdmanii* USDA 3471^T^ [71]*, M. intechi* BD68^T^ [79]*, M. loti* DSM2626^T^ and *M. sanjuanii* BSA136^T^ [77]. All L2 ICESyms, in addition to forming a closely related group based on ANI comparison, carried the additional *nod* genes *nodA2* and *nodFEG,* which were absent in L1 ICESyms. Previous phylogenetic analysis of *nodC* genes of R7A, MAFF303099 and NZP2213^T^ identified a similar pattern of relatedness to that observed in our ICESym symbiotic region ANI [80]. Lineage 3 (L3) of the Loteae ICESyms was solely composed of GII ICESyms and represented a divergent group, with an ANI ≤94 % with L1 and L2, and greater than 97 % amongst one another (Fig. 4). Within L3, ICESyms of NZP2037, SU343, and ATCC 700743^T^ shared ANI values of 100%, reflecting their isogenic nature. However, the tripartite ICESym of NZP2042 and monopartite ICESym of NZP2014 shared 98 and 97% ANI with this isogenic group; hence there were three distinct ICESyms represented in L3 of the Loteae ICESyms (Fig. 4). Given the synteny shared between ICESym representatives of the three Loteae-ICESym lineages (Fig. 3), and the pattern of relatedness observed using ANI comparison (Fig. 4), it appears that the three Loteae-ICESym lineages have diverged from a common ancestral ICESym.

### Putative host-range determinants conserved in broad-host-range GII ICESyms

**Fig. 5.**
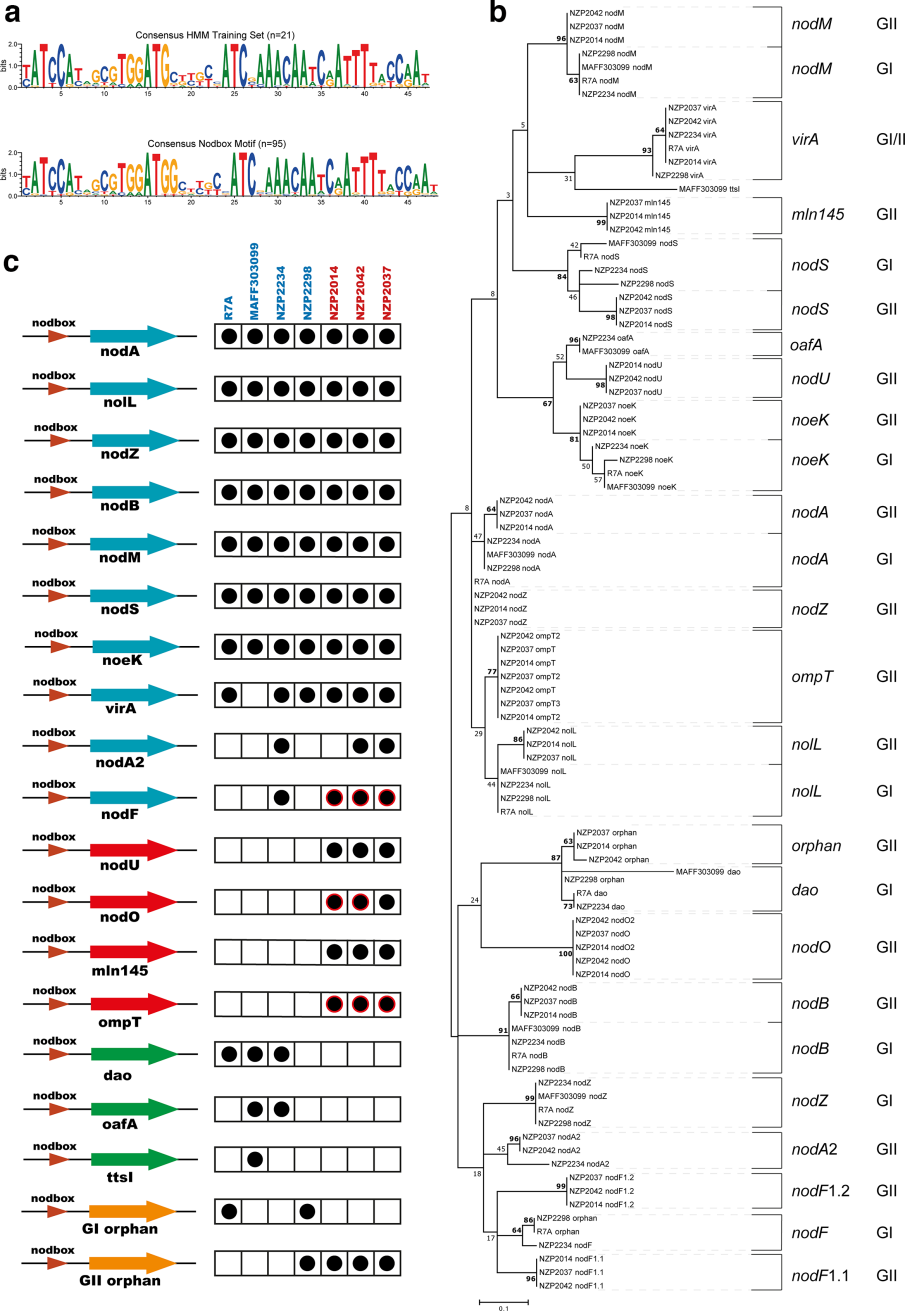
HMM analysis of *nod* box regulatory motifs of GI and GII ICESyms. (a) Consensus *nod* box motif of the HMM training set and the *nod* box motifs identified in the GI and GII ICESym regions. (b) Maximum-likelihood phylogeny with 1000 bootstraps of the 95 *nod* box motifs identified. Bootstrap values greater than 60 are indicated in bold. (c) Overview of *nod* box motif presence or absence in the *nod* box regulons of unique GI and GII ICESyms. Duplicated or triplicate *nod* boxes within an ICESym are indicated with red borders.

Transfer of the GII ICESyms from the *Mesorhizobium* strains NZP2037, NZP2042, SU343 and NZP2014 into a non-symbiotic derivative of the GI strain R7A, R7ANS [81], produced *Lotus*-nodulating strains with host ranges matching those of the GII ICESym donors [54] – confirming that genes carried by the GII ICESyms are responsible for the observed GI/GII host-range differences. To identify conserved protein-coding sequences unique to GII strains, which may be responsible for their expanded host range, two pangenome analyses were conducted, one using complete genome annotations and the other using only ICESym annotations. An 85 % AAi threshold was chosen for all pangenome and pan-ICESym calculations using Roary, as higher thresholds produced inflated accessory genome sizes in both calculations (Fig. 1). As the putative host-range determinant(s) that allow GII strains to nodulate *L. pedunculatus* are ICESym encoded, we focused on genes which were conserved amongst all complete GII ICESyms and were absent from all complete GI ICESyms. The analysis identified 70 such putative genes (Fig. 1, Table S1). Of these, 17 were open reading frames uniquely predicted by Prokka, absent from the genome annotations of NCBI PGAP, and could not be assigned a COG functional category, indicating that they were likely not protein-coding sequences. The remaining 53 protein-encoding genes belonged to 11 different COG groupings, the largest of which was ‘S - Function Unknown’, which contained 21 genes (Fig. 1d). Additional analysis of symbiotic regulatory motifs (discussed in detail below) indicated that within the 53 GII conserved genes, seven were preceded by a *nod* box regulatory motif individually or as part of putative operons, indicating that they may be induced in response to plant flavonoids (Fig. 5c). Six of the seven genes – *nodU* (carbamoyl transferase), the *nodO-prsDE-mln031* operon [type I secretion system (T1SS) and T1SS effector proteins], and *ompT1* (omptin outer membrane protease) *–* were previously identified as present on the GII ICESym of NZP2037 and absent from those of the GI R7A and MAFF303099 ICESyms [4].

The remaining GII ICESym conserved gene, *mln145*, encoded a protein containing Ca^2+^-binding RTX (Repeat in ToXin) domains indicating it may be a T1SS effector protein [82]. Amino acid alignment of the three putative T1SS effectors (NodO, Mln031 and Mln145) revealed that each contained unique regions in addition to the RTX domains, suggesting they may have distinct functions (Fig. S2). The *R. leguminosarum* T1SS effector NodO can support nodulation of strains producing suboptimal NF lacking decorations that affect host range. NodO forms cation-selective channels in lipid bilayers, which led to the suggestion that it may amplify NF-induced Ca^2+^ influx during early stages of symbiotic signalling [83]. Our identification of three T1SS effectors raises the possibility that a cocktail of T1SS effectors, secreted by GII ICESyms in response to plant flavonoids, may act to broaden the host range of *Mesorhizobium* strains harbouring a GII ICESym.

Both GI and GII ICESyms contained a three-gene cluster preceded by a *nifA*-regulated promoter, which consists of a hypothetical DUF683-containing gene followed by *fdxB-syrA* [84]. The SyrA protein has previously been implicated in the post-translational regulation of exopolysaccharide (EPS) production in *Sinorhizobium meliloti* [85]. Through examination of break points in synteny between GI and GII ICESyms, we identified a paralogous copy of the *fdxB-syrA* (*fdxB2-syrA2*) cluster integrated 253 bp downstream of *nodB*. This suggests that expression of *fdxB2-syrA2* may be controlled by NodD via *nodB* regulation. Given the possible roles of EPS in symbiotic signalling in the *Lotus-Mesorhizobium* symbiosis [86, 87], flavonoid-mediated induction of *syrA2* expression may function to alter symbiotic signalling via EPS regulation in GII strains.

All GII ICESyms had two homologues of an OmpT outer-membrane protease, with the ICE*Ml*Sym^2037^ group (ICESyms of ATCC 700743^T^, SU343 and NZP2037) containing an additional third copy. Of the three OmpT paralogues, only Mln327 was conserved at greater than 85 % AAi across all the GII ICESyms. All copies of *ompT* were preceded by a putative *nod* box. Additionally, the three paralogues all contain the crucial Ser-99 catalytic residue [88] as well as the predicted N-terminal Sec-type secretion signal and cleavage site. The divergence of the paralogous copies of OmpT may indicate that these proteins are undergoing rapid evolution. The conserved *ompT nod* box motifs diverge from the canonical *nodA nod* box by a single-base deletion at position 21 between the two conserved regions of the motif. In an *R. leguminosarum nod* box plasmid reporter system, a single base deletion between the proximal and distal *nod* box conserved motifs relaxes NodD repression of the *nodD nod* box in the presence of naringenin, but inhibits induction [15]. This may indicate that the *ompT* genes are not induced by activated NodD. However, it is possible that NodD recognition and induction at *nod* boxes in *Lotus* ICESyms differs from that in the *R. leguminosarum* plasmid reporter system.

### Amino acid variation within NodD1 and NodD2 Nod factor biosynthesis regulators are concordant with host-range groupings

Inspection of amino acid sequence alignments and Mauve alignments of the GI and GII ICESyms indicated that all ten ICESyms contained two distinct conserved homologues of NodD, NodD1 and NodD2, within syntenic *nod* genes clusters. To investigate possible differences in plant flavonoid recognition and subsequent *nod* gene induction between GI and GII ICESyms, we aligned and compared their NodD regulatory proteins. Interestingly, the alignments showed that the NodD1 and NodD2 homologues diverged concordantly with host-range group (Fig. S3). Within the HTH DNA binding domain of the NodDs, the GI and GII NodD1 proteins had a conserved E20A substitution, while the NodD2 proteins had a conserved D46N substitution in the third alpha helix. No substitutions were observed in the second alpha helix, the predicted DNA recognition helix of the HTH domain [89]. The high degree of conservation across the HTH DNA binding domains of the NodD proteins suggests that the affinity of the NodD proteins for a given *nod* box motif would likely be similar.

Within the ligand-binding domain of the NodD proteins, five and eight conserved amino acid substitutions were identified between the GI and GII NodD1 and NodD2 homologues, respectively (Fig. S3). Very little is known regarding the roles of individual amino acids within the ligand binding domains of NodD proteins. In *Sinorhizobium meliloti *, D135 of NodD1 was predicted to function as an acceptor of a hydrogen bond with the inducer luteolin [90], and a D135N substitution abolished induction by luteolin [91]. This residue differs between the GI and GII NodD1 proteins, which may indicate a difference in their ability to interact with specific flavonoids. It has been observed that NodD1 and NodD2 in *M. japonicum* R7A respond to plant inducers at varying stages of infection and, because of reduced conservation between the NodD1 and NodD2 ligand binding domains, it was suggested they may be activated at varying levels by different flavonoids [92].

### Heterogeneity and divergence of *nod* box motifs suggests differences in Nod factor pools synthesized by GI and GII ICESyms

Given that the recognition helices of NodD1 and NodD2 HTH DNA binding domains are conserved between GI and GII ICESyms, differences in the expression levels of *nod* genes may largely reflect sequence variation in the *nod* box motifs preceding them. To identify *nod* box motifs of GI and GII ICESyms, an HMM was trained using 21 previously annotated *nod* boxes from *M. japonicum* R7A and *M. loti* NZP2037 [3, 4] and used to survey the GI and GII genomes for all putative *nod* box motifs. The ICESyms of R88B, SU343 and ATCC 700743^T^ genomes were excluded as they were isogenic with either R7A or NZP2037. The HMM identified 95 putative *nod* box motifs across the seven unique ICESyms, some of which were previously unidentified (Fig. 5b, Table S2). Sequence alignment and clustering of the motifs partitioned them into 21 *nod* box clades with conserved proximal genes, supported by a bootstrap value of greater than 60 – a relaxed threshold from the conventional 70 given the short length of the motifs and the fact that a single substitution in a regulatory motif may have significant impact on its function (Fig. 5b, c).

Comparison of the presence or absence and distribution of *nod* box motifs within the sequence similarity dendrogram identified differences in the NodD symbiotic regulons of GI and GII ICESyms at two levels. Firstly, *nod* boxes were identified that were uniquely conserved in GI or GII ICESyms and, secondly, divergences were observed in *nod* box motifs preceding *nod* genes conserved across GI and GII ICESyms (Fig. 5b). As described above, four conserved GII *nod* boxes were identified that were absent from all GI ICESyms and preceded genes uniquely conserved amongst GII ICESyms, including *nodU*, *nodO*, *mln145* and *ompT* (Fig. 5c, Table S2). This indicates that GII ICESyms have additional branches to their NodD regulons that may be involved in their broad host range. Within the GI ICESyms, three *nod* boxes were observed that were absent from all GII ICESyms; however, none of these were conserved across all GI ICESyms (Fig. 5c). This heterogeneity suggests that these branches of the GI ICESyms NodD regulons are not responsible for the inability of GI strains to nodulate *L. pedunculatus*.

Identification of *nodA2* and *nodF nod* boxes and associated *nod* genes in the GI ICESym of NZP2234, as well as in the ICESym symbiotic regions of *M. erdmanii* USDA 3471^T^ [71], *M. intechi* BD68^T^ [79], *M. loti* DSM 2626^T^ and *M. sanjuanii* BSA136^T^ [77] indicates that L2 Loteae ICESyms (Fig. 4) likely produce NF containing an unsaturated fatty acid modification. This may prove valuable in further dissection of nodulation signalling in *L. japonicus* Gifu, as L2 Loteae ICESyms lack the additional conserved genes found in the broad-host-range GII ICESyms (L3 Loteae ICESyms).

Divergence between the *nod* box motifs identified upstream of *nod* genes present on both GI and GII ICESyms (*nodZ, nodA*, *nolL*, *nodB*, *nodM, nodS* and *nodF*) could indicate differential regulation by NodD (Fig. 5b). The most striking example of this was observed in the divergence of *nod* boxes proximal to *nodZ*, a NF fucosyl transferase implicated as a host-range determinant for nodulation of *L. corniculatus* and *L. filicaulis* by *M. japonicum* R7A [8]. In GI ICESyms, the *nodZ nod* box was similar to the *nodF* and *nodA2 nod* boxes of GII ICESyms; while the *nodZ nod* box of GII ICESyms was significantly different, and most similar to the *nodA nod* box of the primary *nod* gene operon, *nodACIJ-nolO*. Indeed, the GII *nodZ nod* box was identical to the *nodA nod* box of R7A (Fig. 5b). This suggests that the proportion of fucosylated NF may differ between the NF pools produced by GI and GII strains. Alternatively, given that in *M. japonicum* R7A NodD1 and NodD2 induce *nod* genes at differing stages of infection of *L. japonicus* Gifu [92], the significant divergence of GI and GII *nodZ* motifs may indicate that *nodZ* is induced at different stages of infection by GI and GII strains. Differences in *nodZ* regulation may therefore contribute to the differing host ranges of GI and GII strains.

Novel ‘orphaned’ *nod* box motifs were identified, which did not precede any annotated open reading frames. For GI ICESyms, these orphan *nod* boxes clustered with the *nodF nod* box unique to the GI strain NZP2234. The GII orphan *nod* boxes clustered near a novel GI-specific *nod* box group located upstream of a putative d-amino acid oxidase (DAO) gene present in three of the four GI ICESyms (Fig. 5b). The GI strain NZP2298 possessed an intermediate orphan *nod* box motif which clustered between the GII orphan and the GI DAO *nod* box groups (Fig. 5b). Retention of these orphan *nod* boxes at varying locations across the ICESyms suggests that, as in the symbiotic plasmids of *S.fredii* NGR234 [93], horizontal transfer of genetic information adjacent to *nod* box motifs functions as a mechanism for genes which enhance symbiotic fitness, or broaden host ranges, to evolve symbiotic regulation via NodD.

### Summary of GI and GII ICESym core gene function

Pangenomic analysis of the ten complete GI and GII ICESyms identified 155 ICESym-Core genes, three conserved predicted regulatory RNAs (Table S3), and 1740 accessory genes. ICESym-Core genes comprised 30.1 % (±4.8 % sd) of genes found on a given ICESym, while accessory genes represented 63.1 % (±5.2 % sd) of genes. Within each unique ICESym, 34.6 % (±7.7 % sd) of genes were unique to that ICESym, indicating substantial recombination has occurred within the ICESym accessory genes (Table S4). The ratio of accessory to core genes in the GI and GII ICESyms averaged 2.21, while the ratio for the *Mesorhizobium* genomes was 0.78. This inflation of the accessory gene complement in ICESyms relative to that of their host genomes suggests that ICESyms have more relaxed evolutionary constraints than their bacterial hosts with respect to gene acquisition and loss.

The 155 ICESym-Core genes of GI and GII ICESyms represents a subset of the 165 genes previously identified as common between the ICESyms of R7A, MAFF303099 and NZP2037 [4]. Comparison of 14 complete *M. ciceri* ICESyms identified 100 ICESym-Core genes [78]. Variation in the number of ICESym-Core genes identified in different studies likely reflects a combination of differing similarity thresholds used for comparisons and the numbers and phylogenetic range of the ICESyms compared. Our comparison of GI and GII *Lotus* ICESyms used 85 % AAi as the threshold for protein homology as this value gave a high degree of scrutiny without inflation of the pangenome size (Fig. 1).

Within the GI and GII ICESym-Core genes are genes known to be required for ICESym excision, transfer and integration [81, 94], synthesis of NF and nitrogen fixation [3, 84]. Thiamine, biotin, nicotinate and pantothenate biosynthesis genes [95] were also conserved across the ICESyms (Table S3). Interestingly, a nine-gene cluster of gibberellin biosynthesis genes known to influence nodule number and thought to be unique to microsymbionts that form determinant nodules [96] were conserved as was an ACC deaminase [97], indicating that the ability to manipulate plant hormone signalling is a conserved feature of ICESyms found in *Mesorhizobium* strains that nodulate *Lotus*. The three predicted regulatory RNA loci conserved across GI and GII ICESyms included a Thi-box riboswitch, TPP, upstream of *thiC* (*mis389*); a cobalamin riboswitch upstream of *metE* (*msi160*); and a sRNA downstream of a *mucR* regulator (*msi163*) predicted to be a homologue of the ⍺-proteobacterial small RNA ⍺r14. In *S. meliloti *, an ⍺r14 homologue mutant had an impaired symbiotic phenotype [98].

Of the 155 ICESym-Core genes, 90 have been previously characterized or have obvious nodulation or nitrogen fixation related annotations. Thirteen of these genes appear to be unique to *Mesorhizobium* strains that nodulate *Lotus* spp*., Anthyllis vulneria* and *Acmispon wrangelianus* (syn. *Lotus wrangelianus*) – all members of the Loteae tribe of legumes [34]. Both *A. vulneria* and *A. glaber* form indeterminate nodules with mesorhizobia [99, 100]. Furthermore, mesorhizobia that nodulate *A. vulneria* also nodulate *L. corniculatus* [101]. The finding that these 13 genes are not found in mesorhizobia that nodulate other host legumes suggests that they may adapt these ICESyms to plant hosts of the Loteae tribe (Table S3).

## Conclusions

Completion of GI and GII *Lotus*-nodulating *Mesorhizobium* genome sequences allowed extraction of contiguous ICESym regions for exhaustive structural and pangenomic analysis. Comparison of their ICESyms using a host-range framework identified a pattern of evolutionary relatedness, which described the origin and possible genetic basis for the expanded host range conferred by GII ICESyms, which was not apparent from comparison of their complete genome sequences. It appears that the GI and GII ICESyms share a common ancestor from which the ICESym backbone and core genes are inherited. This ancestral Loteae ICESym appears to have radiated into three distinct lineages, as identified by *in silico* DNA–DNA hybridization of ICESym symbiotic regions extracted from 43 Loteae-nodulating *Mesorhizobium* strains. The ancestral ICESym of L2 Loteae ICESyms appears to have acquired *nodA2* and *nodFEG*, nodulation genes required for the synthesis and addition of an unsaturated fatty acid tail to their NF molecules. The ancestral ICESym of the broad-host-range GII ICESyms (L3 Loteae ICESyms) acquired a combination of accessory *nod* genes and recombined *nod* boxes, some of which expanded its host range to confer nodulation of *L. pedunculatus*.

## Supplementary Data

Supplementary material 1

Supplementary material 2
