# Supplementary material for: Symbiosis islands of Loteae-nodulating Mesorhizobium comprise three radiating lineages with concordant nod gene complements and nodulation host-range groupings
Source: Microb Genom. 2020 Aug 26;6(9):mgen000426. doi: 10.1099/mgen.0.000426 (PMC7643969; doi:10.1099/mgen.0.000426)
Supplement: Supplementary material 1 [file mgen-6-426-s001.pdf]

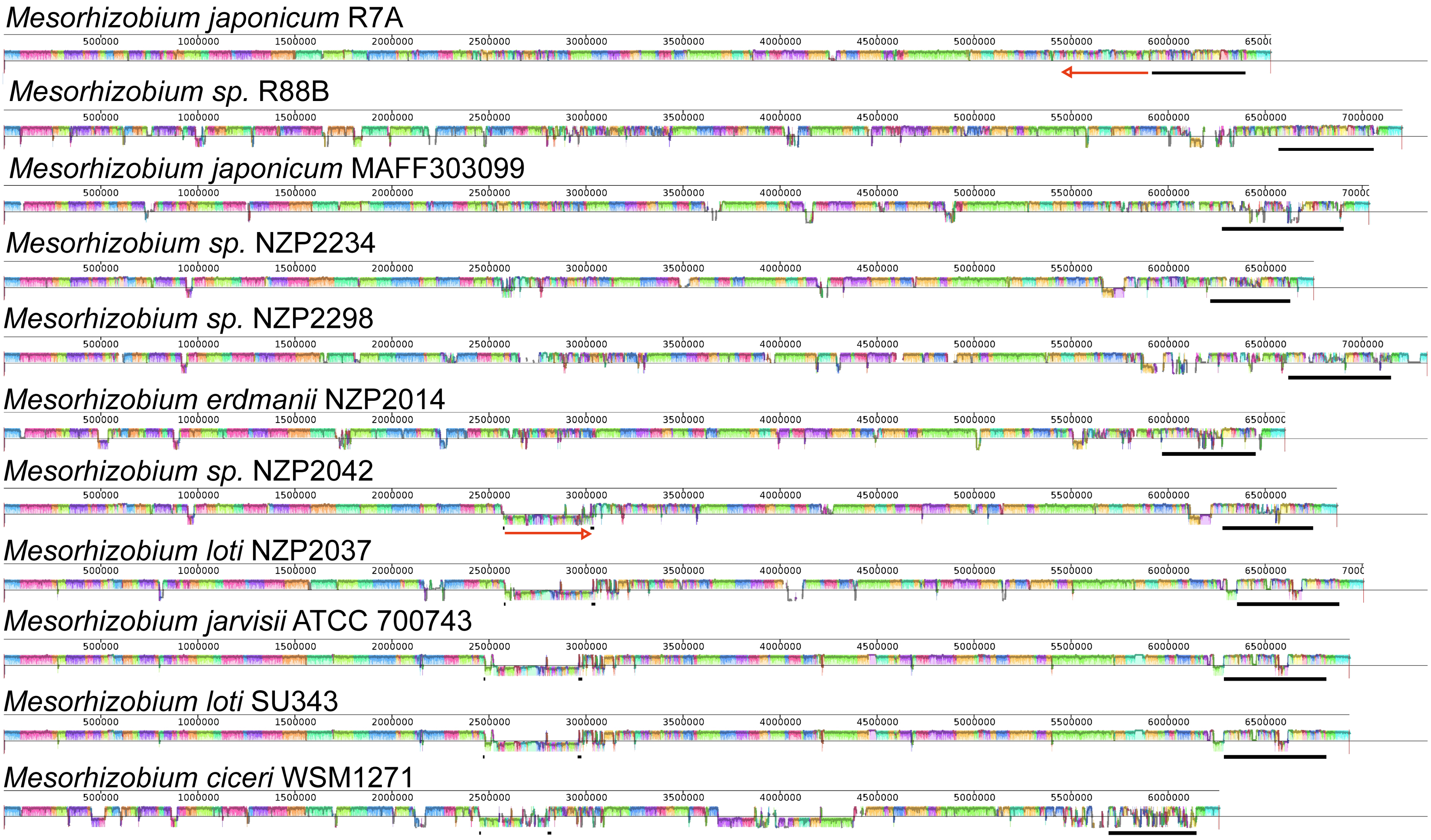


**Figure S1. Shared synteny between GI and GII Mesorhizobium chromosomes.** Ordered coloured regions indicate LCBs present in 2 or more sequences; regions without colour indicate strain-specific sequence; LCBs below the axis indicate regions inverted relative to the M. japonicum R7A chromosome. Regions underlined with black bars indicate the locations of the ICESym regions. Orange arrow indicates the region of chromosomal inversion and translocation in tri-partite ICESym-containing strains relative to M. japonicum R7A. Mesorhizobium ciceri WSM1271 is included as a non-Lotus-nodulating example.

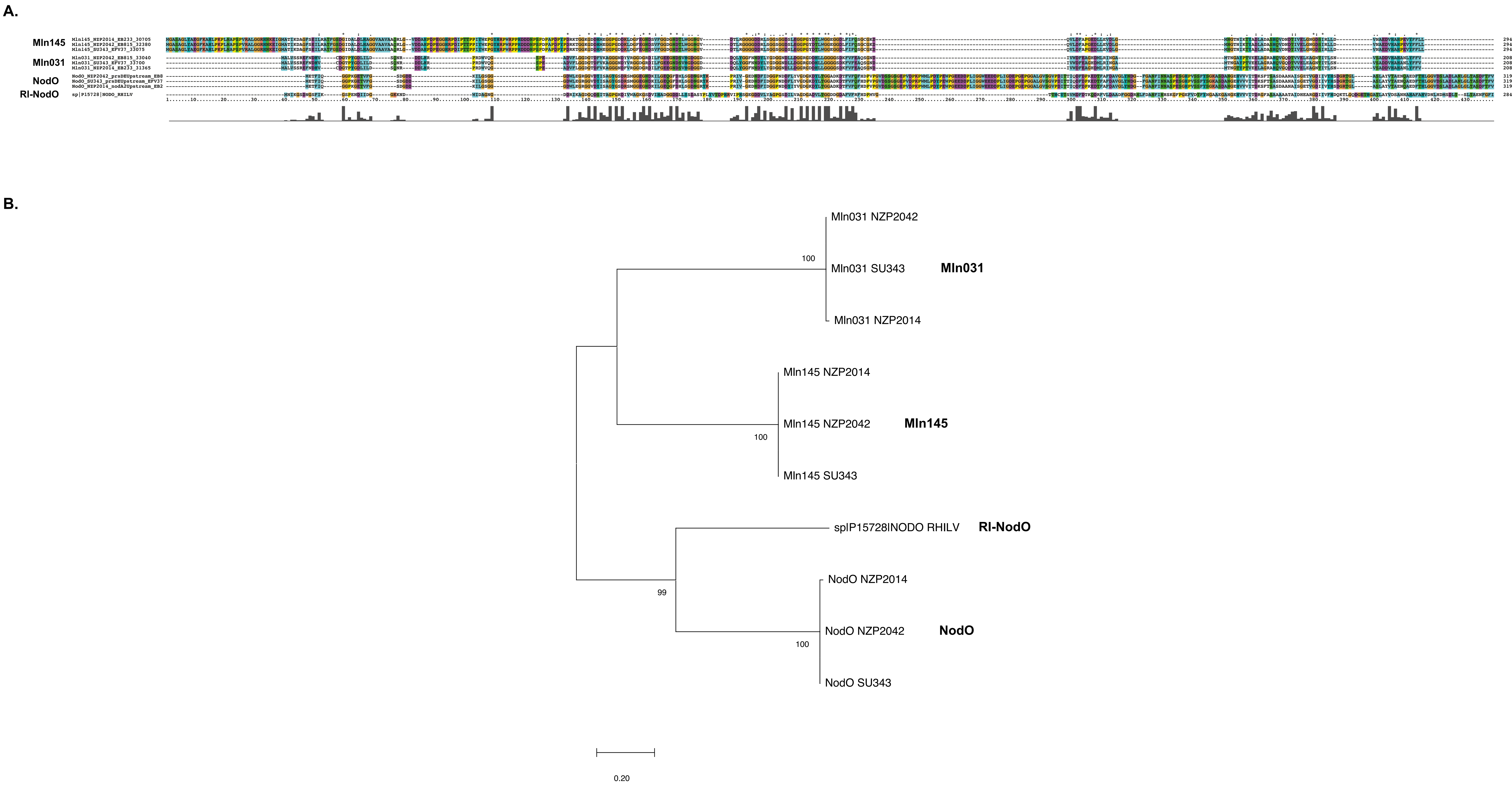


**Figure S2. Amino acid alignment of GII unique type I secretion system effectors.** (A) Alignment of Mln145, Mln031, NodO, and R. leguminosarum NodO proteins. (B) Bootstraped (1000) maximum-likelihood tree of aligned T1SS effectors rooted at the midpoint of the phylogeny

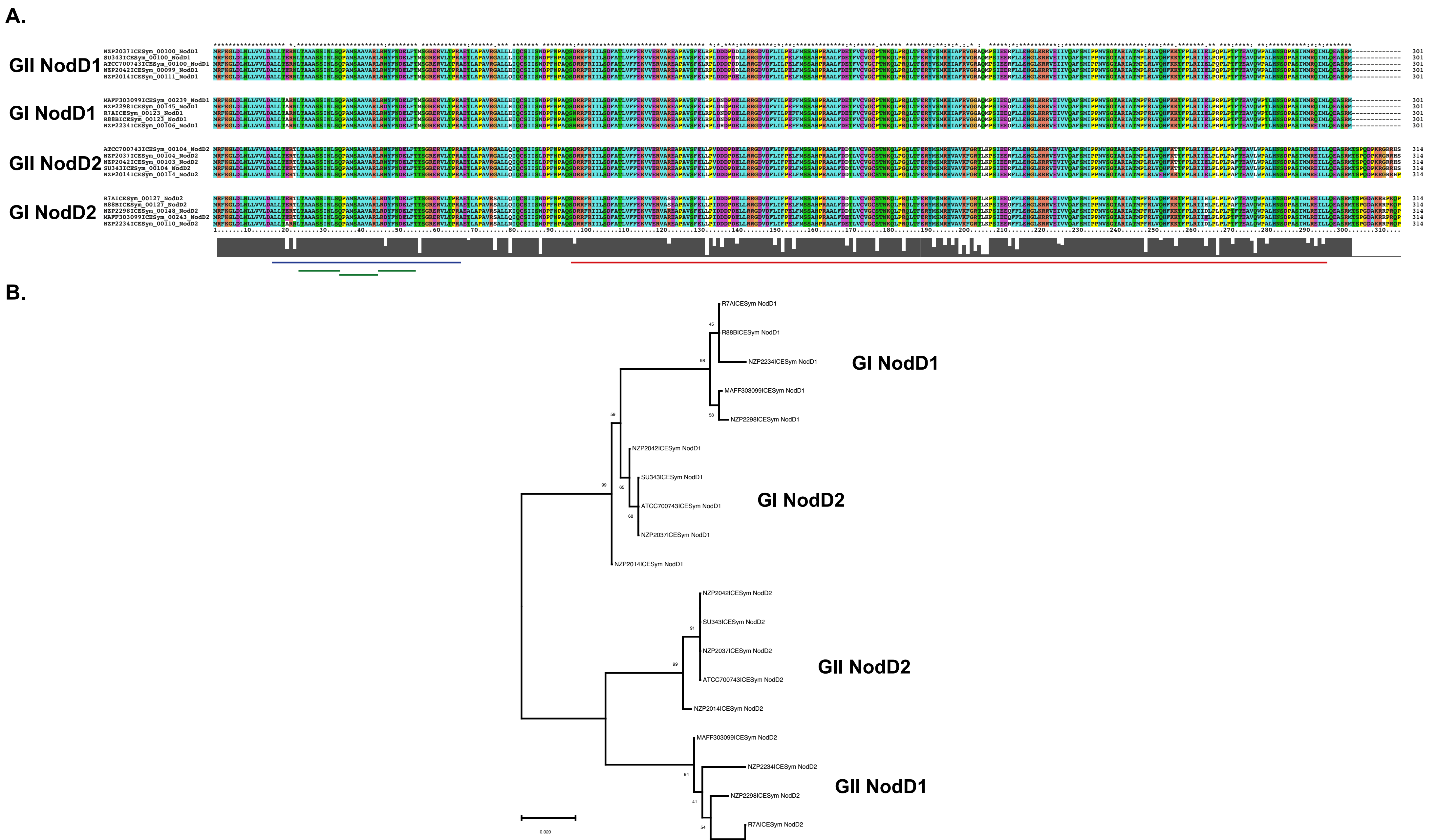


**Figure S3. Amino acid alignment of NodD1 and NodD2 symbiotic regulators.** (A) Alignment of NodD1 and NodD2 from the ten ICESyms compared. Helix-Turn-Helix binding domain is underlined in blue, LysR substrate-binding domain is underlined in red. (B) Bootstraped (1000) maximum-likelihood tree of aligned NodD proteins rooted at the midpoint of the phylogeny.
